# A Lysozyme Murein Hydrolase with Broad-Spectrum Antibacterial Activity from Enterobacter Phage myPSH1140

**DOI:** 10.1128/aac.00506-22

**Published:** 2022-08-11

**Authors:** Nachimuthu Ramesh, Prasanth Manohar, Kandasamy Eniyan, Loganathan Archana, Sudarsanan Athira, Belinda Loh, Long Ma, Sebastian Leptihn

**Affiliations:** a Antibiotic Resistance and Phage Therapy Laboratory, School of Biosciences and Technology, Vellore Institute of Technology, Vellore, Tamil Nadu, India; b Zhejiang University-University of Edinburgh (ZJU-UoE) Institute, Zhejiang University, Haining, Zhejiang, People’s Republic of China; c Fraunhofer Institute for Cell Therapy and Immunology (IZI), Department of Antimicrobial Biotechnology, Leipzig, Germany; d Department of Infectious Diseases, Sir Run Run Shaw Hospital, Zhejiang University School of Medicine, Hangzhou, People’s Republic of China; e University of Edinburgh Medical School, Biomedical Sciences, College of Medicine & Veterinary Medicine, The University of Edinburgh, Edinburgh, United Kingdom

**Keywords:** endolysins, peptidoglycan hydrolase, Gram-negative pathogens, phage therapy, antimicrobial proteins

## Abstract

Bacteriophages and bacteriophage-derived peptidoglycan hydrolases (endolysins) present promising alternatives for the treatment of infections caused by multidrug resistant Gram-negative and Gram-positive pathogens. In this study, Gp105, a putative lysozyme murein hydrolase from Enterobacter phage myPSH1140 was characterized *in silico, in vitro* as well as *in vivo* using the purified protein. Gp105 contains a T4-type lysozyme-like domain (IPR001165) and belongs to Glycoside hydrolase family 24 (IPR002196). The putative endolysin indeed had strong antibacterial activity against Gram-negative pathogens, including E. cloacae, K. pneumoniae, P. aeruginosa, S. marcescens*, Citrobacter* sp., and A. baumannii. Also, an *in vitro* peptidoglycan hydrolysis assay showed strong activity against purified peptidoglycans. This study demonstrates the potential of Gp105 to be used as an antibacterial protein to combat Gram-negative pathogens.

## INTRODUCTION

Antibiotic resistance is a global health issue of major concern. Because of the rapid spread of resistance, new antimicrobial agents are needed and those showing both, broad-spectrum effects and low levels of resistance, would be beneficial (1). Gram-negative bacteria (GNB) are known to cause the majority of serious infections in humans (2, 3). Causative agents that predominantly belong to the *Enterobacteriaceae* family include Escherichia coli, Klebsiella pneumoniae, Enterobacter cloacae, but also other nonfermenting GNB such as Pseudomonas aeruginosa and Acinetobacter baumannii. These bacterial pathogens have been declared a priority by the WHO, with the Disease Society of America creating the acronym “ESKAPE” pathogens that comprise Enterococcus faecium, Staphylococcus aureus, Klebsiella pneumoniae, Acinetobacter baumannii, Pseudomonas aeruginosa and Enterobacter species. These bacteria are known to cause the most critical infections in humans and the development of treatment possibilities is urgent (4–6). Enterobacter species include E. cloacae, *E. hormaechei*, *and E. asburiae* some of which cause infections of the urinary tract but also bloodstream infections (7, 8). Globally, infections caused by antibiotic-resistant Enterobacter are concerning; therefore, antibiotic alternatives are urgently needed.

Phage therapy may be an effective solution to overcome the antibiotic crisis. This is due to the availability, selective killing ability, and low toxicity of phages together with an often-observed synergy with antibiotics (9–14). However, using “living viruses” may not be accepted by the general population due to unfounded concerns as the concept might generate anxiety and lead to the rejection of such a treatment. Phages produce bactericidal proteins that might be a valuable alternative to both, phages and chemical antibiotics. Endolysins are phage-derived enzymes that, during lysis, aid in the release of phage particles from the bacterial host by disintegrating the peptidoglycan (PG) component of the bacterial cell wall (15–17). Endolysins are among the most promising phage-derived protein candidates to fight bacterial infections. While endolysins work by breaking down peptidoglycan of the host cell wall from within, the enzyme can also act from the outside, with studies demonstrating their rapid antibacterial activity, however mostly in the case of Gram-positive pathogens. Another advantage is that endolysins have a broader host range compared to phages, while still exhibiting a narrow activity spectrum compared to chemical antibiotics thus leaving the host’s microflora intact (18–21). Also highly promising is the observation that no resistance to endolysins has been reported thus far (18). Over the past few years, endolysins have received wide-ranging attention as therapeutic agents for the clinic (19–21). Several clinical studies have demonstrated the efficacy of endolysins to eliminate bacterial infections caused by multidrug-resistant pathogens (22, 23).

Enterobacter phage myPSH1140 was isolated against a clinical strain of E. cloacae, which was reported to infect four different species of Enterobacter (24). The genome size was found to be 172,614 bp encoding 102 functional proteins and 138 hypothetical proteins (NCBI accession number: MG999954). In this study, a lysozyme murein hydrolase (Gp105) has been identified in the phage and was first characterized *in silico*, revealing the protein to contain a T4-type_lysozyme domain that belongs to the Glycosyl hydrolase family 24. This protein family is so far uncharacterized in bacteriophages infecting Enterobacter species. In order to evaluate the potential of the protein to be used as a therapeutic, the coding gene was cloned and overexpressed in E. coli. Subsequent tests using the purified protein, which could be obtained in high yield, showed that Gp105 is able to hydrolyze purified peptidoglycan *in vitro* and exhibits strong antibacterial properties against a wide range of pathogens, including E. cloacae, K. pneumoniae, P. aeruginosa, S. marcescens, *Citrobacter* sp., and A. baumannii.

## RESULTS

**FIG 1:**
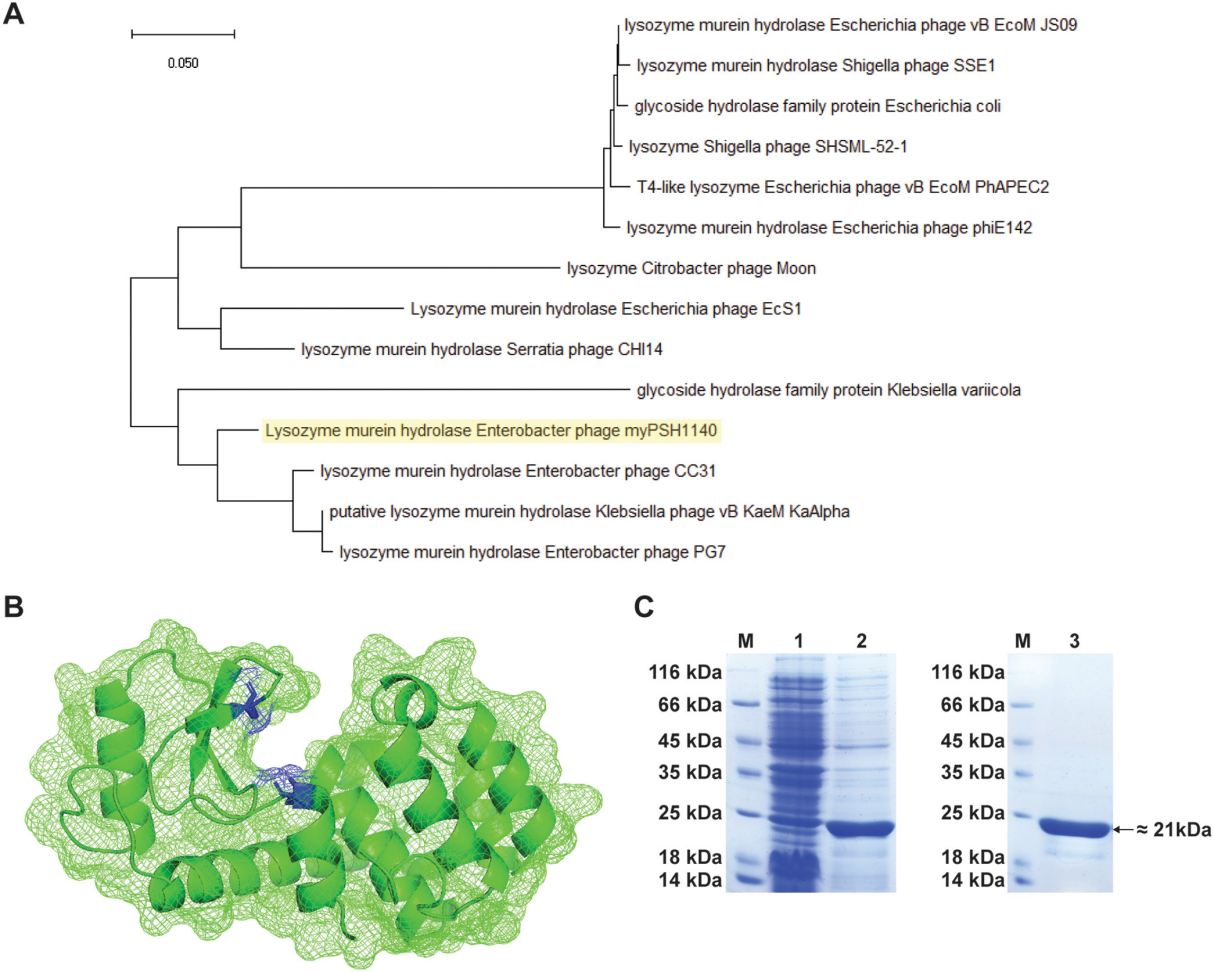
Figure 1A Phylogenetic analysis of Gp105 protein. The sequences were aligned using Clustal Omega Program (https://www.ebi.ac.uk/Tools/msa/clustalo/) and a neighbor-joining tree for conserved sites was built using MEGA software. Figure 1B. AlphaFold2.0 calculated molecular surface model of Gp105 shows a tunnel-like active site topology. The proposed catalytic residues are shaded in blue. Figure 1C. SDS-PAGE analysis of Gp105 protein. Expression of Gp105 protein. Lane 1, not induced. Lane 2, induced. Lane 3, purified. M, protein marker.

In this study, we identified and characterized an endolysin from the genome of the previously characterized Enterobacter phage myPSH1140 (24). The open reading frame Gp105 encodes a 164 amino acid long protein, with a putative lysozyme murein hydrolase function. Such proteins facilitate the release of phage progeny particles from the host bacteria by degrading the peptidoglycan layer, leading to the disintegration, or lysis, of the cells. Phylogenetic analysis revealed that similar types of endolysins can be found in other phage genomes such as Enterobacter phage CC31, Enterobacter phage PG7 and Klebsiella phage vB_KaeM_KaAlpha, with the genome of myPSH1140 exhibiting 90% and 92% similarity with Enterobacter phages CC31 and PG7, respectively (24) (Fig. 1A). According to InterProScan, the protein contains a T4-type_lysozyme-like domain (IPR001165), belonging to the Glycoside hydrolase family 24 (IPR002196). Proteins belonging to the Glycosyl hydrolase family contain catalytic glutamate and aspartic acid residues in their active site required for the cleavage of the glycosidic bond from the substrate (25). Therefore, we used AlphaFold2.0 to obtain a model of the structure (Fig. 1B). The predicted structure shows the position of the key catalytic residues Glu11 and Asp20 inside a clamp-like structure with the residues facing each other, allowing the enzyme to “grab” the peptidoglycan and hydrolyze it while sliding along the polymer network.

### Gp105 hydrolyzes purified peptidoglycan *in vitro*.

Since Gp105 exhibits features similar to other endolysins reported previously and contains the key catalytic residues embedded within a predicted structure that make it likely to be able to hydrolyze peptidoglycan, we cloned the gene into an E. coli expression vector. The recombinant protein was produced in E. coli and then purified by affinity chromatography to obtain the N-terminal His-tagged protein with a molecular weight of 21 kD (Fig. 1C).

**FIG 2:**
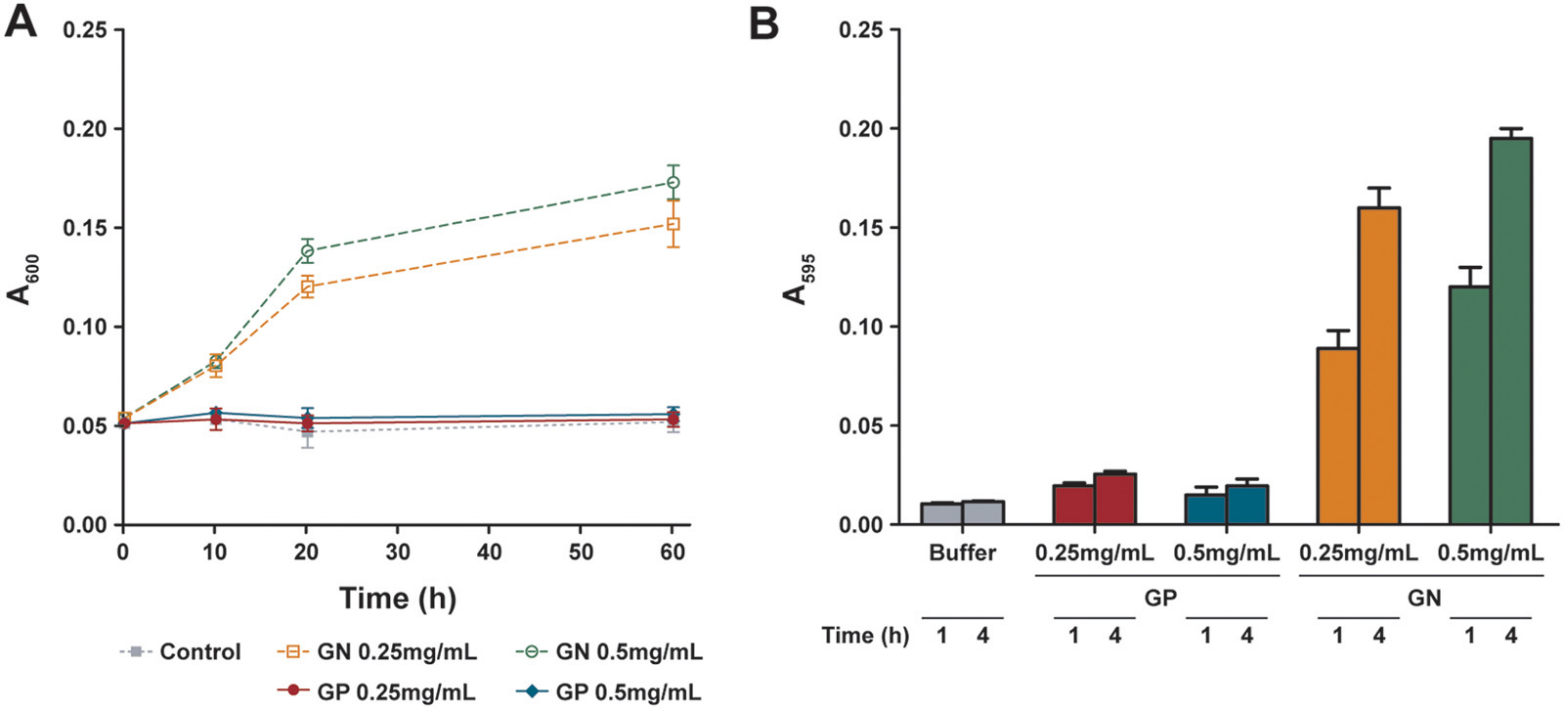
Peptidoglycan hydrolase activity of Gp105 against peptidoglycan isolated from Enterobacter. (A) The peptidoglycan degradation assay was measured using the absorbance (*A_600_*) at time zero (*t_0_*) and time *t* (*ΔA_600_*) and in the control group, the purified peptidoglycan was incubated with buffer. (B) The activity was measured by RBB dye release assay and in the control group, RBB-dye-labeled peptidoglycan was incubated with buffer. GP, Gram-positive bacteria, S. aureus; GN, Gram-negative bacteria, E. cloacae.

To assess if the protein is functional and able to hydrolyze peptidoglycan, we purified peptidoglycan from Enterobacter cloacae following a protocol previously established by Santin et al. (26). Two different assays were then employed; one that makes use of the intrinsic absorbance of the isolated material, while the second one follows the release of the Remazol Brilliant Blue dye (RBB). Using both assays, we could demonstrate that Gp105 can hydrolyze peptidoglycan from Enterobacter. We then tested if the enzyme exhibited a broader activity or if Gp105 is specific for the genus of Enterobacter (Fig. 2A). However, with peptidoglycan isolated from the Gram-positive bacterium S. aureus no activity was observed (Fig. 2B). Thus, endolysin Gp105 shows specificity toward its bacterial host, E. cloacae. Our data demonstrate that Gp105 is indeed a peptidoglycan hydrolase that has Gram-negative pathogens enzymatic specificity.

### Endolysin Gp105 inhibits the growth of Gram-negative bacteria.

In Gram-negative bacteria, endolysins are released into the periplasm after their synthesis, making it necessary to cross the inner membrane. Often, specialized pore-forming proteins, including holins, facilitate this process. The outer membrane is a barrier to endolysins when added to cells from the outside, thus protecting the peptidoglycan layer from degradation. To assess if Gp105 also has antibacterial activity, we performed two tests with 12 Gram-negative bacteria belonging to eight genera, including E. coli, K. pneumoniae, E. cloacae, A. baumannii, S. typhi, P. aeruginosa, S. marcescens and *Citrobacter* sp. in addition to four Gram-positive bacteria (two *Enterococcus* sp. isolates and two S. aureus strains).

In the first assay, we assessed the growth of bacteria on solid media with a reservoir of the endolysin present, slowly diffusing into the agar (Table 1). No inhibition zone was observed in the case of bacterial cells that were not treated or treated with low concentrations of EDTA. Using pretreated cells (2 mM EDTA), the strains were exposed to different concentrations of purified endolysin Gp105 at final concentrations of 0.007 mg/mL to 0.5 mg/mL. Here, clear inhibition zones were observed in the case of the Acinetobacter, Pseudomonas, and *Serratia* strains that were tested and to a slightly lesser extent with the Enterobacter, Klebsiella, and *Citrobacter* isolates. In contrast, we observed that neither the tested Salmonella nor the Escherichia strains showed inhibition zones in this semiquantitative well-diffusion assay, as was also observed in the case of the Gram-positive *Enterococcus* and Staphylococcus isolates.

**TABLE 1:** Antibacterial activity of endolysin Gp105 as observed using well-diffusion and MIC assays^*a*^

Bacteria	Well-diffusion	MIC (mg/mL)
Escherichia coli 1512	−	>0.5
Escherichia coli 1005	−	>0.5
Klebsiella pneumoniae 1199	++	0.25
Klebsiella pneumoniae 1204	++	0.25
Pseudomonas aeruginosa 1528	+++	0.125
Pseudomonas aeruginosa 16	++	0.25
*Enterobacter cloacae 140	++	0.25
Enterobacter cloacae 1507	++	0.25
Acinetobacter baumannii 1505	+++	0.125
Salmonella Typhi 749	−	>0.5
Serratia marcescens 349	+++	0.125
*Citrobacter* sp. 57	++	0.25
*Enterococcus* sp. 501	−	>0.5
*Enterococcus* sp. 774	−	>0.5
Staphylococcus aureus 428	−	>0.5
Staphylococcus aureus 422	−	>0.5

*^a^*Bacterial cells were treated with 2 mM EDTA. The endolysin Gp105 concentrations used were 0.8 mg/mL for well-diffusion assay and 0.007–0.5 mg/mL for MIC. +++, zone size of >10 mm; ++, zone size of <10 mm.

The second test was performed in liquid; similar to the testing of antibiotic compounds, we determined the MIC (MIC) for the Gp105 protein (Table 1). No growth was observed at endolysin concentrations from 0.125 to 0.5 mg/mL in the case of E. cloacae, K. pneumoniae, P. aeruginosa, S. marcescens, *Citrobacter* sp., and A. baumannii (Table 1). Correlating with the results from the diffusion assay, no growth inhibition was observed in the case of E. coli, S. typhi, E. faecalis, and S. aureus in the presence of the maximal concentration of the protein, 0.5 mg/mL.

**FIG 3:**
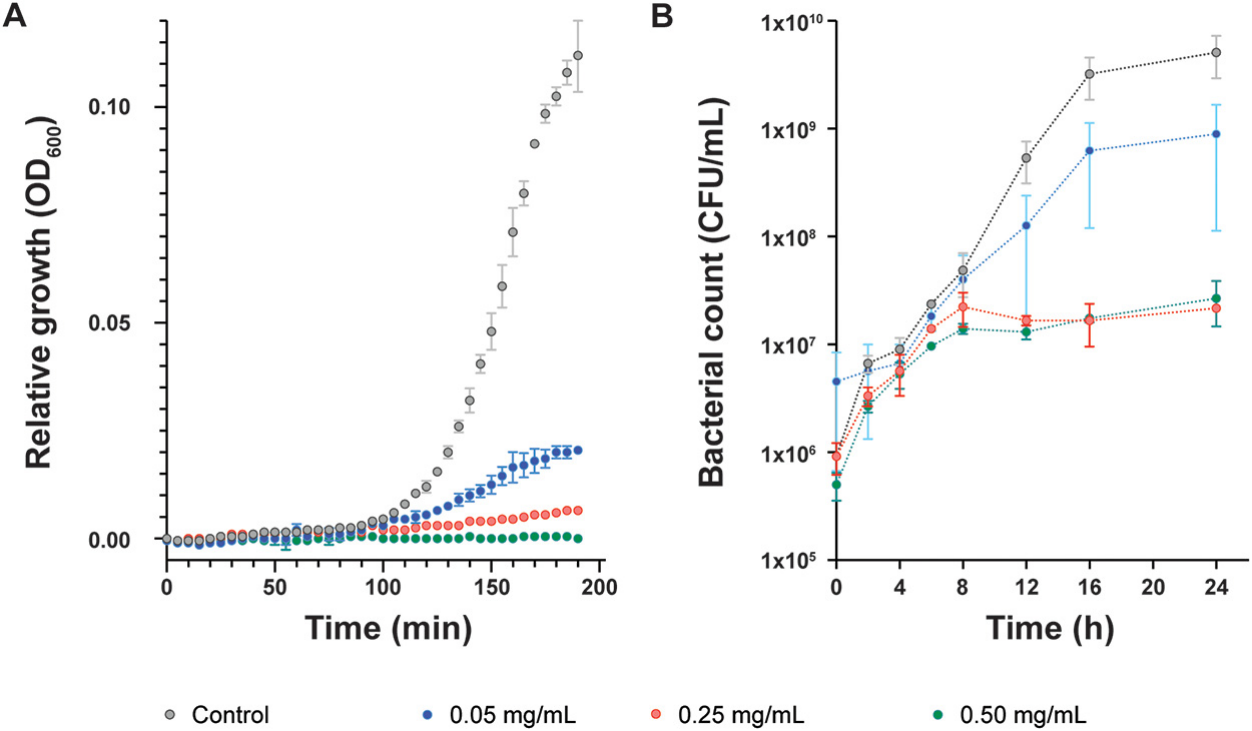
Antibacterial activity of lysozyme murein hydrolase, Gp105 against Enterobacter cloacae. (A) Optical density measurement to determine growth inhibition of E. cloacae pretreated with 2 mM EDTA when exposed to 0.05, 0.25 and 0.5 mg/ml of Gp105 final concentration. At the concentrations used, the protein buffer has no effect on bacterial growth (Supplementary Fig. S1). (B) The viability of cells was assessed by counting the number of CFU (CFU). In the control groups, the bacteria are grown without any antibacterial compound. Data shown are standard mean values obtained from three repeated experiments.

Next, we determined the growth inhibition for E. cloacae at endolysin concentrations of 0.05, 0.25 and 0.5 mg/mL. When measuring the growth by determining the optical density at 600 nm, a significant reduction in bacterial growth was observed within 5 min after the addition of 0.25 and 0.5 mg/mL of Gp105 (Fig. 3A). When counting the CFU at different time points, we were able to obtain live bacteria. However, over time the number did not show any major increase (Fig. 3B). Thus, the protein appears to show bacteriostatic properties, which is unusual for endolysins. A possible explanation might be that the enzyme only acts on dividing cells where it might be able to traverse the outer membrane barrier.

## DISCUSSION

Antibiotic resistance is rapidly developing into a major global health crisis, creating a requirement to identify and develop new treatment options employing chemical or biological antimicrobial compounds. Endolysins as therapeutic proteins are considered to be an effective alternative to treating bacterial infections (27). Based on the specificity of the enzyme, endolysins can be categorized into five main groups: (a) N-acetylglucosaminidases, (b) N-acetylmuramidases, which cleaves the glycan bond found on the reducing side of the MurNAc; these are also called lysozymes, (c) lytic transglycosylases, (d) N-acetylmuramoyl-l-alanine, and (e) peptidases (28).

The endolysin we have investigated is from an Enterobacter phage, underrepresented in published reports. To the best of our knowledge, this is the first study of an endolysin from a phage infecting Enterobacter sp. In this work, the purified protein Gp105 with a predicted lysozyme murein hydrolase function from Enterobacter phage myPSH1140 was found to have broad-spectrum antibacterial activity against Gram-negative bacteria. The hydrolase activity was also confirmed by using peptidoglycans isolated from E. cloacae.

Gp105 is a T4-type_lysozyme domain-containing protein which belongs to the Glycosyl hydrolase family. The T4 lysozyme hydrolyzes the 1,4-beta linkages between *N-acetyl-d*-glucosamine and N-acetylmuramic acid in peptidoglycan heteropolymers of bacterial cells (25). Further studies are required to determine if the mechanism behind the enzymatic activity of Gp105 is the same as that of the T4 lysozyme.

Gp105 does not reduce the turbidity of the culture media containing target cells as we observe only an arrest in the optical density values and not a decrease. This indicates that the exposure of susceptible bacteria to the phage protein does not result in the disintegration of cells even though the bacterium is still inactivated (i.e., killed), as demonstrated by our experiments in which we determined the number of viable cells (Fig. 3B). Similar observations have been made in the past with phages; certain phages do not lead to clear culture solutions (i.e., a reduction in optical density) although the host cells undergo lysis, and release phage progeny (13).

The recombinant expression of Gp105 in E. coli allowed the high-yield production of a functional protein with yields of approximately 1–2 mg of purified protein per mL of culture. The high yield expression is possibly due to the inactivity of the protein toward E. coli, as also shown in the results of the well-diffusion assay. The observed molecular weight of 21 kDa is similar to that of other endolysins previously reported which exhibit sizes between 16 and 25 kDa (29, 30).

Especially promising is the fact that Gp105 shows broad-spectrum activity against at least six Gram-negative pathogens. However, as with all other endolysins acting on Gram-negative bacteria, cells were treated with EDTA which binds and removes divalent ions that stabilize the surface structure of cells, thus resulting in a partially destabilized outer membrane. Other published studies have reported the use of such compounds to study the activity of the enzymes against Gram-negative bacteria (31). While the use of chemicals such as EDTA is medically not possible, the key aspect of studies such as ours and previously reported research is the activity of the enzyme *per se*, albeit in concert with EDTA. Adding stretches of amino acids such as Lysine or Arginine which are oppositely charged to the cell envelope or creating fusion proteins with cell-membrane penetrating peptides, might allow the use of such synthetic enzymes without EDTA in the future. It is also worth mentioning that most of the characterized endolysins are effective in bacterial growth media but inactive in complex media such as serum.

### Conclusion.

In our study, we characterized a lysozyme murine hydrolase, Gp105, from Enterobacter phage myPSH1140 and analyzed its structure and antibacterial activity *in silico*, *in vitro* and *in vivo*. Gp105 is a T4-type lysozyme domain-containing protein that belongs to the Glycosyl hydrolase family. The identified peptidoglycan hydrolase gene was cloned, expressed, and purified to explore its ability to inactivate Gram-negative bacteria. The broad-spectrum activity of Gp105 could be exploited to inhibit or kill pathogenic bacteria in therapeutic applications.

## MATERIALS AND METHODS

### Bacterial strains, plasmids, and chemicals.

Escherichia coli DH5α and Escherichia coli BL21(DE3) were used for cloning and expression studies, respectively. The clinical strains of Escherichia coli, Klebsiella pneumoniae, Enterobacter cloacae, Acinetobacter baumannii, Salmonella Typhi, Pseudomonas aeruginosa, Serratia marcescens, *Citrobacter* sp., *Enterococcus* sp., and Staphylococcus aureus were used from the Antibiotic Resistance and Phage Therapy Laboratory, VIT, Vellore. T4 DNA ligase and restriction enzymes BamHI and XhoI were obtained from New England Biolabs (USA). Oligonucleotide primers were synthesized by Eurofins Scientific India Pvt. Ltd. (India). The plasmid- pET28a was purchased from Novagen (Germany). Ni-NTA agarose was purchased from G-Biosciences (USA), and plasmid isolation and gel extraction kits were obtained from TaKaRa Biotech (USA). All analytical grade chemicals and antibiotics were purchased from Himedia Chemicals (India). Standard recombinant DNA techniques were used as described elsewhere (32).

### Phage propagation and DNA extraction.

The phage enrichment method was used to propagate phage myPSH1140 against the host bacteria, E. cloacae. Briefly, 1 mL of phage lysate was added to 3 mL of overnight grown bacterial cultures (host bacteria), and the mixture was incubated at 37°C for 24 h in a shaking incubator (150 rpm). The mixture was centrifuged at 6000 × *g* for 15 min and the supernatant was filtered through a 0.22-μm syringe filter. The filtrate was tested for phage activity using a spot test and double agar overlay method as previously described (24).

Phage DNA was extracted from the enriched phage lysate using the phenol-chloroform (24:1) method. Briefly, the phage particles were treated with DNase (20 mg/mL) and RNase (0.5 mg/mL) for 1 h at 37°C to remove any host DNA and RNA. To the mixture, 20% PEG and 1.6 M NaCl were added and stored at 4°C for 1 h. The content was centrifuged at 12,000 × *g* for 10 min and to the pellet, phage lysis buffer (50 μL of 10% SDA, 50 μL of 0.5 M EDTA, and 5 μL of 10 mg/mL proteinase K) was added, mixed well and incubated overnight at 50°C. An equal volume of phenol: chloroform: isoamyl alcohol (25:24:1) was added and centrifuged at 12,000 × *g* for 15 min (repeated twice). The aqueous phase was extracted into a new tube and an equal volume of 100% isopropanol was added to precipitate the DNA at −20°C for 6 h. The precipitated DNA was pelleted, washed with 100% ethanol and air-dried. The purified phage DNA was visualized on 0.8% agarose gels.

### PCR amplification.

The nucleotide sequence of the gp105 gene was retrieved from NCBI (http://www.ncbi.nlm.nih.gov) for designing gene-specific primers. PCR was performed using the genomic DNA of Enterobacter phage myPSH1140 as a template. The gene was amplified using gene-specific primers listed in table S1, containing the BamHI (Forward primer) and XhoI (Reverse primer) restriction sites. The gene was amplified under the following conditions: 95°C for 5 min, 25 cycles (95°C 1 min; 55°C 1 min; 72°C 1 min), and a final extension of 72°C for 10 min. The PCR products were analyzed on a 1.2% agarose gel.

### Cloning and expression.

The PCR products generated were gel purified, digested, and ligated into the pET28a vector. The ligation mixture was transformed into E. coli DH5α cells and the colonies obtained were screened using T7 Promoter and T7 Terminator primers (table S1). The recombinant plasmid was isolated from the positive colonies using a plasmid miniprep kit and verified by DNA sequencing.

The recombinant plasmid pET28a-gp105 was transformed into E. coli BL21(DE3) cells for expression studies. A single colony of E. coli BL21(DE3) containing pET28a-gp105 was inoculated in 5 mL of LB medium containing kanamycin (50 μg/mL) and grown at 37°C overnight. 1 mL of this culture was added to a 100 mL fresh LB medium containing kanamycin (50 μg/mL) and incubated at 37°C to reach the log phase. To the growing culture of E. coli BL21(DE3) containing pET28a-gp105, IPTG was added to a final concentration of 1 mM and incubated at 37°C for 3 h. After induction, cells were harvested by centrifugation at 8,000 × *g* for 10 min and the total protein was analyzed (both the control and induced cells) in 12% SDS-PAGE. The proteins were visualized by staining the gel with Coomassie blue (0.05%) for 30 min in a rocking shaker.

### Protein purification.

E. coli BL21(DE3) cells transformed with pET28a-Gp105 were grown at 37°C in LB broth containing kanamycin (50 μg/mL) until the log phase and the expression was induced by adding 1 mM IPTG for 3 h at 37°C. The cells from the 100 mL culture were resuspended in 5 mL of lysis buffer (50 mM phosphate buffer, pH 8.0, 300 mM NaCl, 5 mM imidazole, 1 mM PMSF and 10 μg/mL lysozyme). After one freeze-thaw cycle, the cells were lysed by sonication (30s pulse × 30s pause). The soluble fraction was separated by centrifugation at 15,000 × *g* for 30 min at 4°C and loaded onto a prepacked 2 mL (bed volume) Ni–NTA agarose column. After washing the column with 30-bed volumes of Wash buffer A (50 mM Phosphate Buffer, pH 8.0, 300 mM NaCl, 20 mM imidazole) and Wash buffer B (50 mM Phosphate Buffer, pH 8.0, 300 mM NaCl, 40 mM imidazole) at a flow rate of 1.0 mL/min, the bound protein was eluted in Elution buffer (50 mM phosphate buffer, pH 8.0, 300 mM NaCl, 200 mM imidazole). The purified proteins were quantified using the Bradford assay.

### Peptidoglycan purification.

The bacterial peptidoglycan (PG) from Enterobacter cloacae and Staphylococcus aureus was obtained as described earlier by Santin et al. (26). Briefly, 400 mL of bacterial cells at OD_600_ 1 to 1.2 were harvested by centrifuging at 10,000 × *g* for 20 min at 4°C. The bacterial pellet was resuspended in 20 mL of buffer I (20 mM Tris-Cl and 100 mM NaCl, pH 8). The bacterial cells were broken by passing (3 to 5 times) through a syringe needle (0.4- by 20 mm needles) and the cell wall was collected by ultracentrifugation at 90,000 × *g* for 45 min at 4°C (Beckman Coulter, Inc.). To the pellet, 10 mL of 8% SDS was added and incubated at 96°C for 1 h. The peptidoglycan fraction was collected by ultracentrifugation at 90,000 × *g* for 45 min at 25°C and the obtained peptidoglycan was resuspended in 10 mL of 0.5 M NaCl and 10 mL of 8% SDS. The peptidoglycan was mixed thoroughly and incubated at 96°C for 30 min. The peptidoglycan was then pelleted by ultracentrifugation at 90,000 × *g* for 30 min at 25°C and resuspended (twice) in 10 mL of sterile distilled water. Then, the peptidoglycan was resuspended in 10 mL of buffer II (20 mM Tris-HCl, pH 7.2, 50 mM NaCl supplemented with 200 μg/mL α-amylase and 200 μg/mL pronase) and incubated at 37°C overnight. After incubation, 10 mL of 8% SDS was added and incubate for an additional 1 h at 96°C. The peptidoglycan was pelleted by ultracentrifugation at 90,000 × *g* at 25°C for 30 min and the obtained peptidoglycan fraction was resuspended (twice) in 10 mL of water. The pellet obtained in the final step was resuspended in 1 mL of water. The peptidoglycan was stored at 4°C until use.

### Peptidoglycan hydrolysis assay.

The hydrolysis of peptidoglycan at different concentrations of endolysin Gp105 was determined using a peptidoglycan hydrolysis assay (26). Briefly, 125 μL of purified peptidoglycan was diluted with 875 μL of 60 mM MES (pH 6), and 180 mM NaCl and incubated at 37°C for 30 min. The peptidoglycan was then incubated with different concentrations (0.05, 0.25 and 0.5 mg/mL) of endolysin Gp105 at 37°C and the absorbance at 600 nm was measured at regular time intervals. The peptidoglycan hydrolysis was measured by plotting the difference between absorbance at the time (t) and initial absorbance (t0).

### Colorimetric peptidoglycan hydrolysis assay.

The peptidoglycan was labeled with Remazol Brilliant Blue (RBB-PG) as described earlier by Uehara et al. (33) and the peptidoglycan hydrolysis was determined based on the colorimetric assay. Briefly, to 10 μL of RBB-PG, 90 μL of PBS buffer was added and incubated at 37°C for 30 min. After incubation, 0.05, 0.25 and 0.5 mg/mL of endolysin Gp105 were added to the mixture and incubated for up to 6 h (hydrolysis period). The reaction was quenched by adding 100 μL of absolute ethanol after every 1 h or hydrolysis period. The release of RBB dye was measured by pelleting the peptidoglycan at 90,000 × *g* at 25°C for 30 min and the absorbance (A_595nm_) was measured. The graph was plotted using the absorbance measured after 1 and 4 h when the RBB-labeled peptidoglycan was incubated with buffer and endolysin Gp105.

### Antibacterial assay.

The anti-bacterial assay was performed as described previously (34). This assay was performed in the presence or the absence of outer membrane permeabilizers, i.e., EDTA. Briefly, the bacteria (E. cloacae) was grown in LB broth at 37°C to reach log-phase culture. The bacterial cells were collected by centrifugation at 6000 × *g* for 10 min and washed once with sterile distilled water. For EDTA treatment, the bacterial pellet was resuspended in LB broth and EDTA was added to 2 mM, and incubated for 5 min at 37°C. After incubation, the cells were centrifuged at 6000 × *g* for 10 min and washed twice with sterile distilled water. Then, the bacterial pellet was resuspended in LB broth and used for further experiments, with controls that were not treated with EDTA but otherwise processed identically. Well-diffusion assay was performed on LB agar plates. Here, 200 μL of up to 0.8 mg/mL of endolysin was added into mechanically excised holes, “punched” wells (radius: 1 mm) and incubated at 37°C for 16 h. The inhibition zone, where no growth was observed, was recorded.

A time course analysis of antibacterial activity was performed to determine the reduction of bacterial cell numbers over time. Briefly, log-phase bacterial cells (E. cloacae treated with 2 mM EDTA) were incubated with 0.05, 0.25 and 5 mg/mL of endolysin Gp105 in LB media at 37°C for 24 h. The optical density was measured at 600 nm at every 5 min interval for 4 h. In parallel, a 100 μL aliquot of bacterial cells was removed at 0, 2, 4, 6, 8, 12, 16, and 24 h, followed by determining the number of cells (CFU) by plate counting. All the experiments were repeated three times for statistical significance.

### Determination of broad-spectrum activity.

To test the broad-spectrum antibacterial activity of purified Gp105 protein, a total of 12 Gram-negative bacteria belonging to eight genera were chosen. This includes E. coli, K. pneumoniae, E. cloacae, A. baumannii, S. typhi, P. aeruginosa, S. marcescens, and *Citrobacter* sp. The four Gram-positive bacteria include two *Enterococcus* sp. and S. aureus.

The MIC of endolysin Gp105 was determined to analyze the activity. Accordingly, the microbroth dilution method (34) was followed in which the purified endolysin Gp105 was used at concentrations ranging from 0.007 mg/mL to 0.5 mg/mL. The endolysin Gp105 activity was determined against the log-phase bacterial culture treated with EDTA (2 mM as determined from the previous experiment). Any reduction in the bacterial turbidity was noted and the minimum concentration at which no observable bacterial growth was determined was the MIC.
