# Supplementary material for: A Lysozyme Murein Hydrolase with Broad-Spectrum Antibacterial Activity from Enterobacter Phage myPSH1140
Source: Antimicrob Agents Chemother. 2022 Aug 11;66(9):e00506-22. doi: 10.1128/aac.00506-22 (PMC9487488; doi:10.1128/aac.00506-22)
Supplement: Supplemental file 1 — Table S1 and Fig. S1. Download aac.00506-22-s0001.pdf, PDF file, 0.5 MB [file aac.00506-22-s0001.pdf]

Supplemental table 1:

Table 1. The primer sequences were used to amplify the gene, Gp105.

Primer Name	Sequence Details (5’- 3’)**
Gp105-Forward	AACGGATCCGAGGTCCTCATGGAC
Gp105-Reverse	CGCCTCGAGATAACCGCCTGGAACTCGC
T7-Promoter	TAATACGACTCACTATAGGG
T7-Terminator	CTAGTTATTGCTCAGCGGTG

** Restriction sites in the primer sequences are underlined.

Supplementary Figure 1

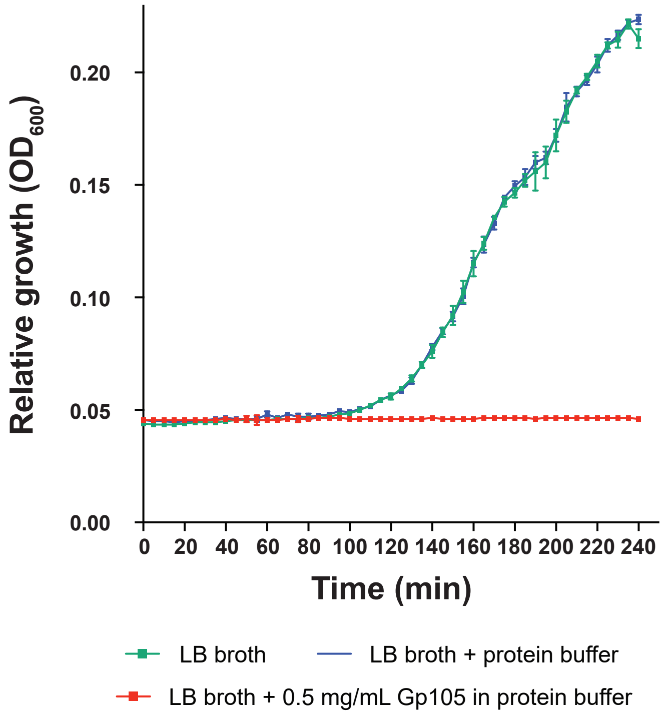

Supplementary Figure 1.The protein buffer (containing imidazole) has no effect on bacterial growth at concentrations we used in our experiments. LB broth: Lysogeny Broth
